# Cytogenomic characterization of 1q43q44 deletion associated with 4q32.1q35.2 duplication and phenotype correlation

**DOI:** 10.1186/s13039-018-0406-0

**Published:** 2018-11-06

**Authors:** A. M. Mohamed, H. T. El-Bassyouni, A. M. El-Gerzawy, S. A. Hammad, N. A. Helmy, A. K. Kamel, S. I. Ismail, M. Y. Issa, O. Eid, M. S. Zaki

**Affiliations:** 1 Division of Human Genetics and Genome Research, Department of Human Cytogenetics, National Research Centre, 33-El-Bohooth St. Dokki, Cairo, 12311 Egypt; 2 Division of Human Genetics and Genome Research, Department of Clinical Genetics, National Research Centre, Cairo, Egypt

**Keywords:** Chromosome 1q43-q44 deletion syndrome, Chromosome 4 duplication syndrome, Multiple abnormalities, Microcephaly, Hypogenesis of corpus callosum

## Abstract

**Background:**

Microdeletion of 1q43q44 causes a syndrome characterized by intellectual disability (ID), speech delay, seizures, microcephaly (MIC), corpus callosum abnormalities (CCA) and characteristic facial features. Duplication of 4q is presented with minor to severe ID, MIC and facial dysmorphism. We aimed to verify the correlation between genotype/phenotype in a patient with 1q43q44 deletion associated with 4q32.1q35.2 duplication.

**Case presentation:**

We report on a 3 year-old female patient with delayed motor and mental milestones, MIC and facial dysmorphism. She is a child of non-consanguineous parents and no similarly affected family members. CT brain showed abnormal gyral patterns, hypogenesis of corpus callosum and bilateral deep Sylvian fissure. Electroencephalogram showed frontotemporal epileptogenic focus. Her karyotype was revealed as 46,XX,add(1)(q44). Fluorescence in situ hybridization (FISH) using whole chromosome paint (WCP1) and subtelomere 1q revealed that the add segment was not derived from chromosome 1 and there was the deletion of subtelomere 1q. Multiple ligation probe amplification (MLPA) subtelomere kit revealed the deletion of 1q and duplication of 4q. Array CGH demonstrated the 6.5 Mb deletion of 1q and 31 Mb duplication of chromosome 4q.

**Conclusion:**

The phenotype of our patient mainly reflects the effects of haploinsufficiency of AKT3, HNRNPU, ZBTB18 genes associated with duplication of GLRA3, GMP6A, HAND2 genes. Patients presented with ID, seizures, MIC together with CCA are candidates for prediction of 1q43q44 microdeletion and cytogenomic analysis.

## Background

Chromosome 1q43-q44 deletion (OMIM; 612,337) is a characteristic syndrome. Mankinen et al. 1976 were the first to characterize the 1q deletion [1]. Since that time about 150 patients were reported [2–11]. The clinical phenotype is characterized by microcephaly (MIC), growth retardation, facial dysmorphism, corpus callosum abnormalities (CCA), seizures, cardiac, and gastroesophageal and urogenital anomalies [7, 11, 12]. All the patients with terminal 1q deletion have mild to severe intellectual disability (ID), variable degrees of delayed speech and dysmorphic features. The patients have a round face, hypertelorism, low set or malformed ears. The presence of seizures, CCA, and MIC varied according to the involved genes. Several authors tried to recognize the smallest region of overlap (SRO) responsible for the neurodevelopmental delay in 1q terminal deletion particularly for MIC, CCA, and seizures. They recognized AKT3, HNRNPU, CEP170, ZBTB18 (ZNF238) as candidate genes responsible for the neurodevelopmental delay [11–14].

Partial trisomy 4q is an infrequent chromosomal disorder caused by duplication of the distal end of the long arm of chromosome4. Several studies reported the clinical manifestations of chromosome 4 duplication and found that this disorder displays distinctive phenotype, including craniofacial, renal, heart and thumb defects. Patients with duplication of 4q32q35 are presented with MIC/craniosynostosis, hypertelorism, epicanthal fold, thick abnormal eye-brows, downward slanting of palpebral fissure, broad/prominent nasal bridge, low set malformed ears, long philtrum, small chin, and short neck [15–21]. These defects result from an increased dosage of genes located on the duplicated -region particularly the effect of GLRA3, GMP6A, and HAND2 genes [17–21]. Some other authors found the minimal clinical effect of 4q duplication [22–24].

Herein, we report on a female patient with 1q43q44 deletion associated with 4q32.1q35.2 duplication. We aimed to verify the correlation between genotype/phenotype in a patient with 1q43q44 deletion associated with 4q32.1q35.2 duplication.

## Case presentation

**Fig. 1:**
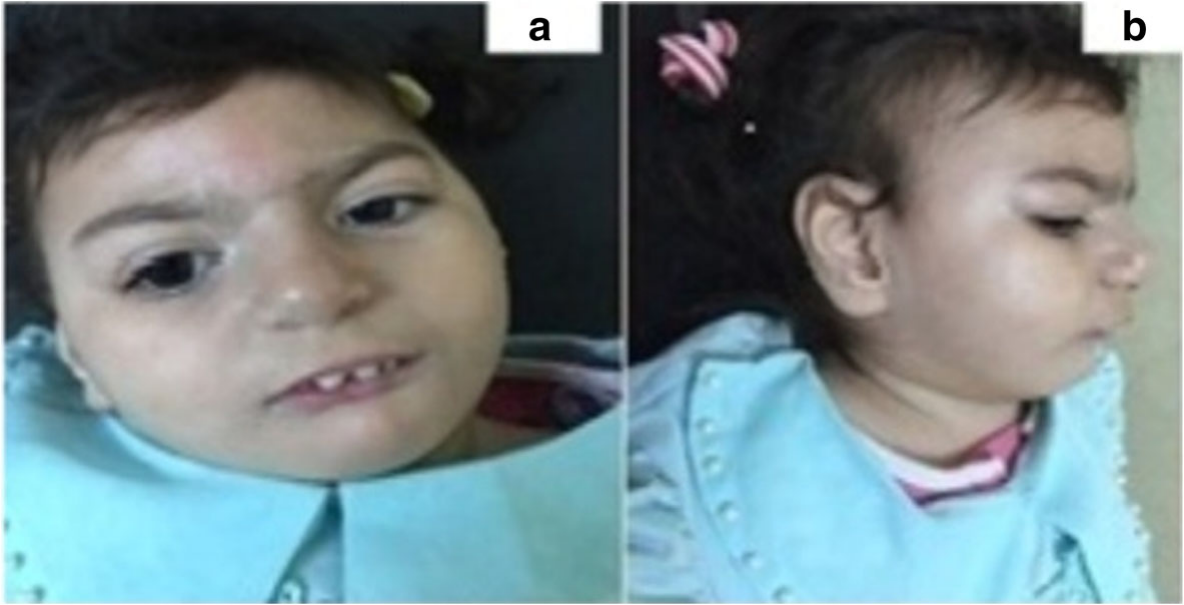
**a** and **b** Facies showing narrow forehead, thick, bow-shaped eyebrows, synophrys, long smooth philtrum, low set ears, retro-micrognathia and short neck

A 3-year-old girl was referred to the neurogenetics clinic, National Research Center, Egypt because of the delayed milestones of development and unusual facies. She was the offspring of a non-consanguineous marriage with no similarly affected family members. The pregnancy and delivery histories were uneventful, however small head and dysmorphic facies were noted at birth. Delayed milestones and failure to gain weight were noted since early life. Seizures were developed at the age of 9 months as myoclonic and the focal seizures were fairly controlled on a combination of valproate and levetiracetam. Evaluation of the motor and mental developmental milestones was remarkably delayed; she could only sit supported, had impaired cognitive functions with obvious autistic features, had the inability to maintain holding objects, and didn’t acquire any speech skills. Her main anthropometric measurements revealed head circumference 40 cm (−6 SD), length 79 cm (−3.6 SD) and weight 7.200 kg (−3 SD). Clinical examination showed dysmorphic facies including, round face with full cheek, narrow forehead, thick bow shaped eyebrows, hypertelorism, long smooth philtrum, downturned corners of the mouth, low set ears, retro-micrognathia and short neck (Fig. 1: a and b). She had bilateral simian creases, vascular markings on the palm, tapering fingers, and clitoromegaly on genital assessment. Neurological evaluation showed hypotonia with elicited reflexes. Table 1 shows a comparison of the main clinical presentation, involving cytobands, size of 1q deletion, smallest region of overlap (SRO) in the previously reported patients with pure 1q43q44 submicroscopic deletion and our patient.

**Table 1:** Comparison of our findings to that of other authors, particularly, in clinical presentations, deleted cytobands, involved genes, the size of the deleted bands as well as the user workstations

	Our patient	Boland et al. 2007	Hill et al. 2007	Van Bon et al. 2008	Caliebe et al. 2010	Zaki et al. 2012	Nagmani et al. 2012	Thierry et al. 2012	Ballif et al. 2012	Parlman et al. 2013	Sisami et al. 2015	Hemming et al. 2016	Raun et al. 2016	Depienne et al. 2017
Number of patients	1	6	7	13 (exclude 2 familial patients)	4	1	7	11	22	1	1	1	1	17 patients + 37 of previous 1q43q44 deletion
Cytoband	1q43q44	1q42q44	1q43q44	1q43q44	1q44	1q43q44	1q43-q44	1q44	1q43q44	1q44	1q43q44	1q43-q44	1q44	1q43q44
Size in Mb	6.5	3.5 Mb	2	0.36	0.44	10.4	1.4	0.63	2	1.47	8	8	4.1	1.36
Genomic location (hg19)	242,664,760-249,206,918	243,500,000–244,750,000	243,000,000-245,000,000	244,533,377-244,833,377	244,968,377-245,394,377	238,681,384-249,190,989	242,987,737-244,331,570	244,900,000-245,100,000	243,433,377-245,433,377	244,125,269-245,594,168	241,178,091-249,224,121	241,183,190-249,202,755	244,842,325-248,938,897	243,100,00-244,500,000
SRO in Mb	6.5	1.25	2 Mb	0.36	0.44	10.4	0.8	0.188 for seizures and ID	2 for CCA,MICand SZR	1.47	8	8	4.1	1.36
Platform	Affymetrix HD array CGH, hg19	aCGH using high resolution BAC-tiling	Microsatellite and SNP	Different Agilant, Affymetrix	Agilent and Illumina	Affymetrix	Custom designed	Agilent. Using custom trgeted Agilent array	Agilent and Roch-NimbleGene	Affymetrix,SNP array 6	Blue genome array CGH	Infinium Human Cyto SNP (Illumina)	105 k CMA oligoV7.2	Different platforms
Genomic Build	hg19	Hg17	hg17	hg18	hg18	hg18	hg18	Hg19	Hg18	hg18	hg19	hg19	hg19	hg19
Involved genes	CEP170, AKT3, ZBTB18, HNRNPU	AKT3,EP171,ZNF238 (ZBTB18)	CEP179, SDCCAG, AKT3	C1orF100, ADSS, C1orF101, PNAS-14	FAM36A, HNRPU, EFCAB2, part of KIF2613	C1or100, ADss, C1orf101, PNAS4	CEP170, ZNF238, SDCCAG8	HNRNPU, FAM36A, NCRNA00201	AKT3, ZNF238, FAM36A, CIORF199, HNRNPU	ZNF238, CEP170	AKT3, ZNF238, FAM36A, HNRNPU	PLD5, CEP170, SDCCAG8, AKT3, ZNF238, HNPNPU	HNRNPU	AKT3, HNRNPU, ZBTB18
ID	+	4 are neonates, and 2 + ve	+	11/11	+	+	+	+ in all patients	+	+	prenatal	+	+	+
CCA (agenesis or partial)	+	In 5 patients	5/7	9/11	4/4	+	+in 4 patients	+ only in patient 2	+ in 7 patients	+	-ve	+	+	in 7 patients
MIC	+	+ in 4 patients	6/7	11/11	+	+	+	+ only in patient 1 and 2	+ in 7 patients	-ve	+	+	+	in 49 patients out of 54
seizures	+	In 3 patients	6/7	9/11	+	+	+in 3	+ in all patients	+ in 9 patients	+	prenatal	+	+	in 36patients out of 54
Dysmorphic features	+	In 5 patients	+	+	+	+	+	+ in all patients	+	+	+	+	+	+

**Fig. 2:**
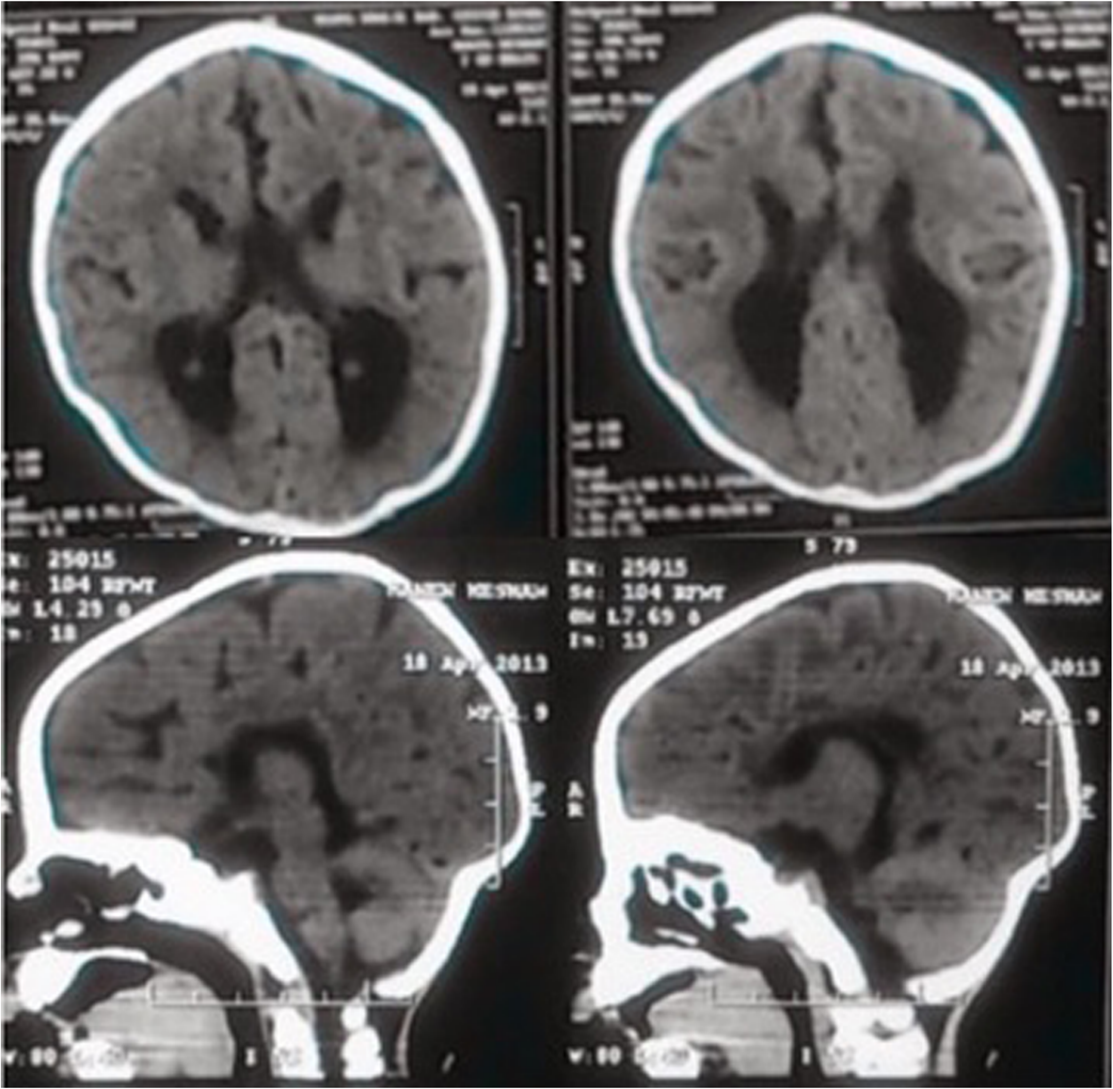
CT brain without contrast, axial (upper row) and sagittal (lower row) showing abnormal simplified gyral pattern, hypogenesis of corpus callosum, colpocephaly and bilateral deep Sylvian fissure

Electroencephalogram showed frontotemporal epileptogenic focus. CT brain displayed abnormal gyral pattern, hypogenesis of corpus callosum and bilateral deep Sylvian fissure (Fig. 2). Echocardiogram, fundus examination, abdominal and renal ultrasonography revealed no abnormalities. Psychomotor assessment using Stanford Binet International Scale method showed profound retardation.

This study was carried out in compliance with the Declaration of Helsinki and approved by the National Research Centre Ethical Research Committee.. Informed consent was obtained from the parents for genetic testing and publication of this case report.

## Methods

GTG banding was performed at 550 band level, karyotype and nomenclature according to the ISCN 2016 [25].

Fluorescence in situ hybridization (FISH) using whole chromosome paint (WCP) of chromosome 1 (Cytocell) and mix 1 of ToTelVysion probe (Abbott) was performed according to the manufacturer’s procedure.

Multiple ligation probe amplification (MLPA) was done using SALSA MLPA probemix P070-B2 Human Telomere-5 according to the manufacturer’s instruction (MRC-Holland). The PCR products were electrophoresed in the ABI 3500 genetic analyzer (Applied Biosystems, USA). MLPA data analysis was performed using the Coffalayser software (www.mlpa.com).

Array CGH was done according to the manufacturer’s manual, and using Cytoscan HD Gene chip (Affymetrix Santa Clara USA), Gene chip hybridization oven 645, wash using fluidic station 450 (Affymetrix), scanned by Gene chip scanner 3000, using chromosome analysis suit (CHAS) software.

## Results

**Fig. 3:**
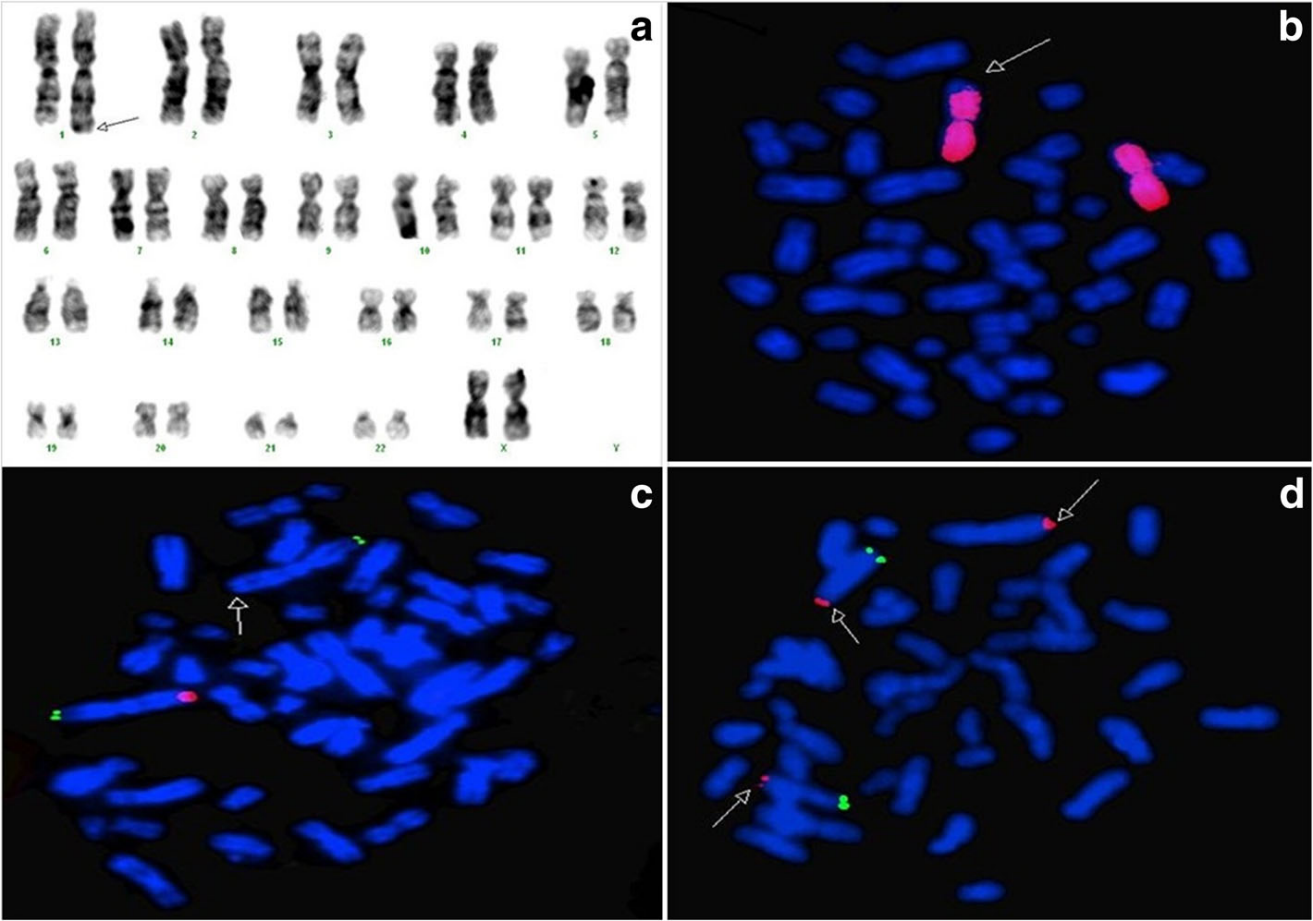
(**a,b,c** and **d**). (**a**). karyotype of the proband revealed 46,XX, add(1) (q44), (**b**). painting probe (WCP) for chromosome No.1(spectrum red) showed that the add segment was not derived from chromosome 1, (**c**). Total Subtelomere Kit (Vysis) using mix No.1 demonstrated the normal two signals of subtelomere 1 p (spectrum green) and only one signal of subtelomere 1q (spectrum red), (**d**).Total subtelomere Kit (Vysis) using mix No.4 demonstrated the normal two signals of subtelomere 4 p (spectrum green) and 3 signals of subtelomere 4q (spectrum red)

Cytogenetics revealed the karyotype that 46,XX,add(1)(q44), (Fig. 3a), both parents had a normal karyotype. Fluorescence in situ hybridization (FISH) using whole chromosome paint 1(WCP) demonstrated that the add segment was not derived from chromosome 1, and using mix 1 of total subtelomere probes (Abbott) demonstrated the deletion of 1q subtelomere (Fig. 3: b and c). Multiple ligation probe amplification (MLPA) showed the subtelomeric deletion at 1q44 and duplication at 4q35.2. The duplicated 4q subtelomere was confirmed by FISH using mix 4 of total subtelomere (Fig. 3:d).

**Fig. 4:**
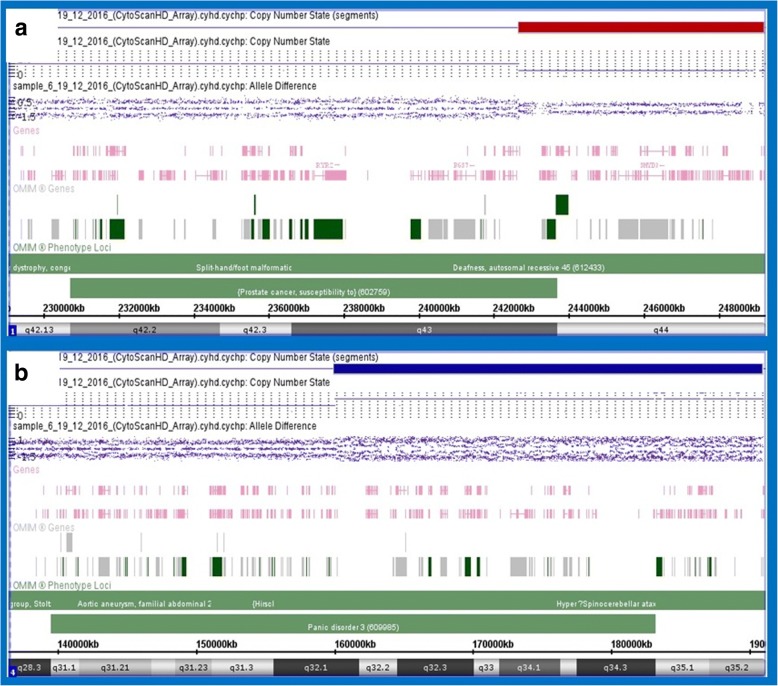
**a**). HD array CGH with 1q deletion, **b**). HD array CGH with 4 qduplication

Array CGH revealed 6.543 Mb loss of chromosome 1 and 30.955 Mb gain of chromosome 4 (Fig. 4: a and b):

46,XX.arr[GRCH37]1q43q44(242,664,760_249,206,918)× 1, 4q32.1q35.2(159,996,280_190,951,473)× 3.

Table 1 compares our findings to that of the other authors particularly in clinical presentations, deleted cytobands, involved genes, the size of the deleted bands as well as the user workstations.

**Fig. 5:**
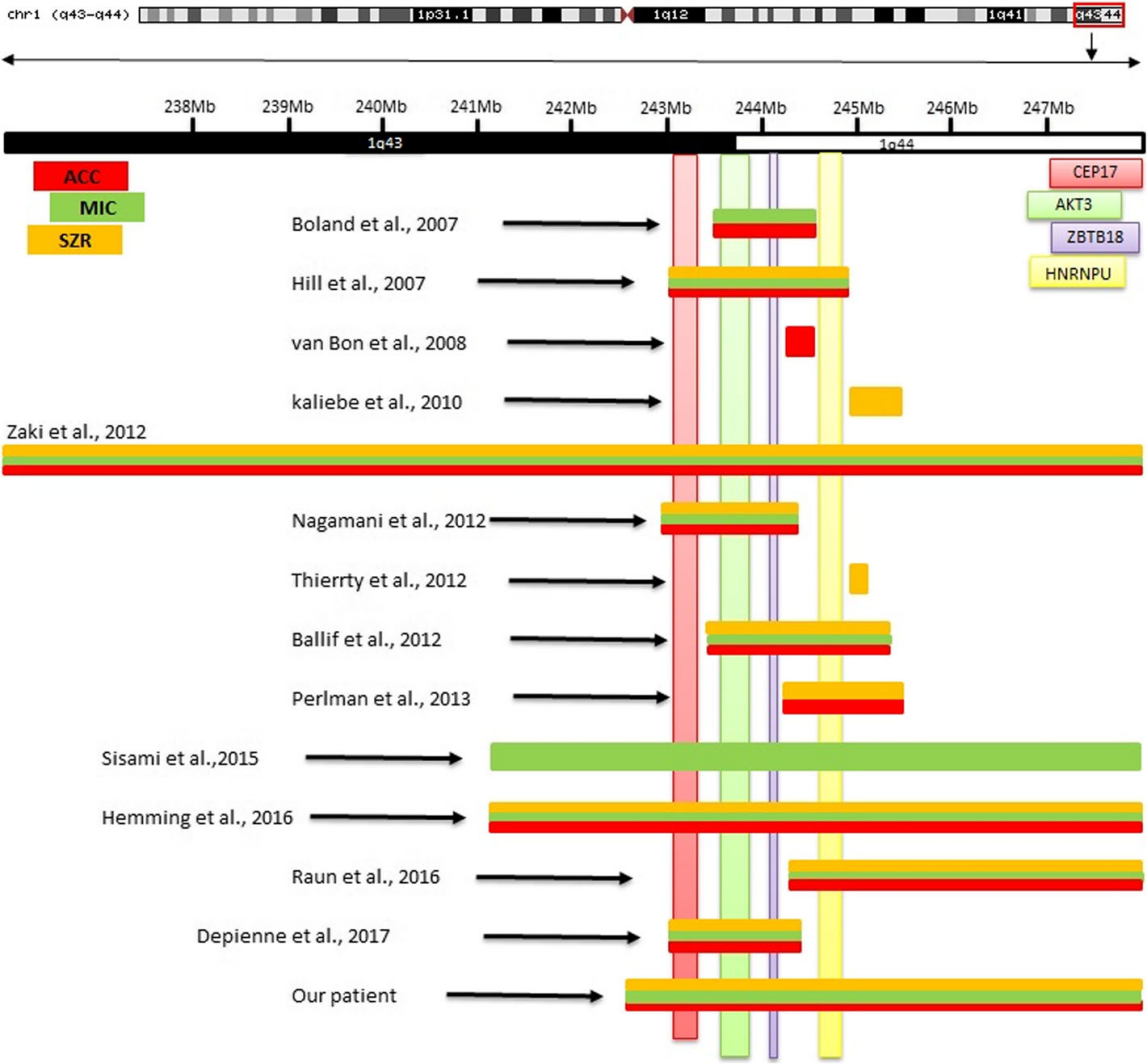
Comparison of the size of the 1q deleted region, involved cytoband and deleted genes in relation to CCA, MIC and seizures in our patient and in other authors studies

Figure 5 shows a comparison of the size of 1q deleted region, involved cytoband and deleted genes in relation to corpus callosum abnormalities (CCA), microcephaly (MIC) and seizures in our patient and some previously described patients with pure 1q43q44 deletion.

**Fig. 6:**
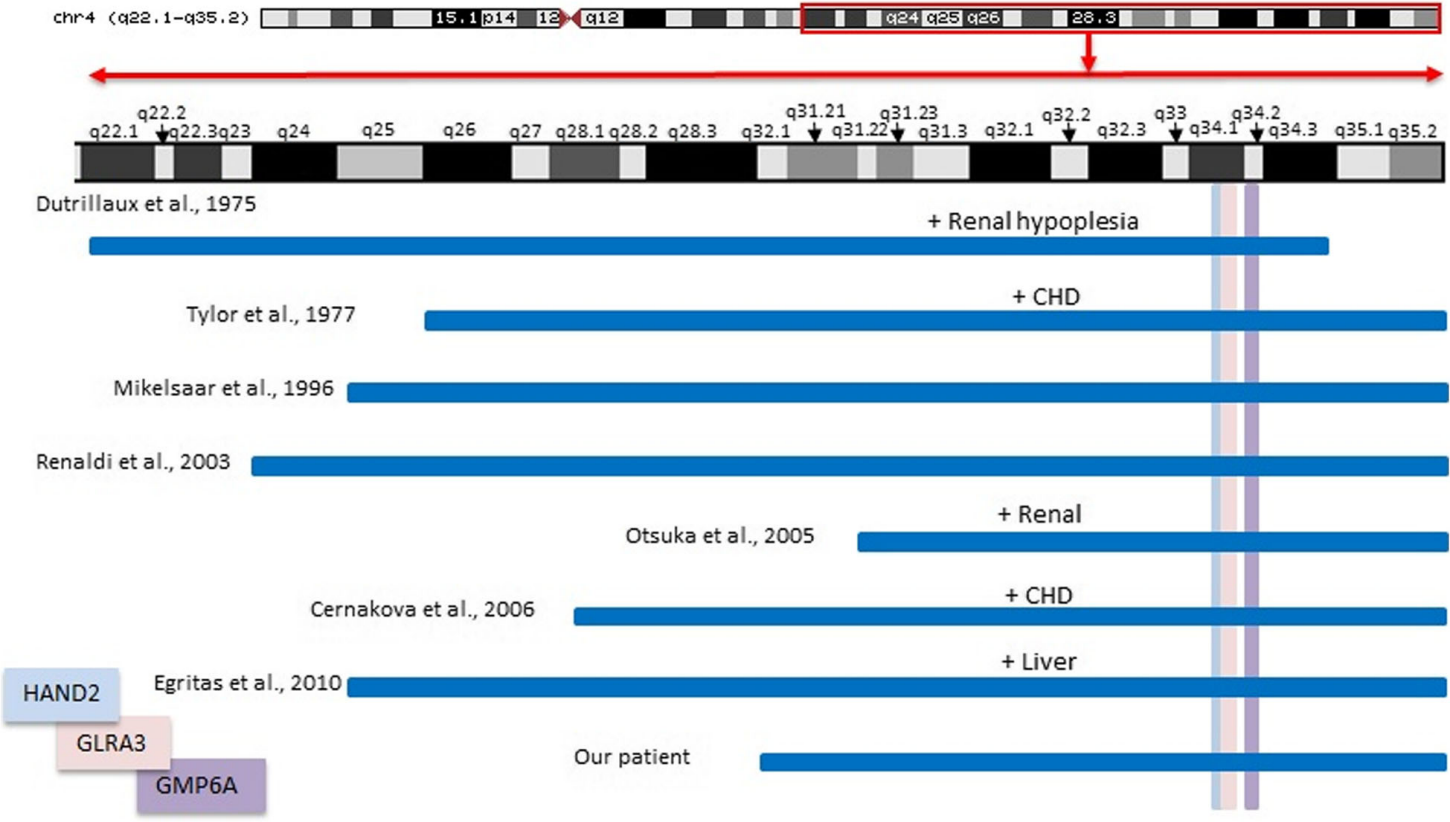
Shows comparison of the size of 4q duplicated region, involved cytoband and duplicated genes in relation to MIC, ID, dysmorphic features and other congenital anomalies

Figure 6 shows a comparison of the size of 4q duplicated region, involved cytoband, and duplicated genes in relation to MIC, ID, dysmorphic features, and other congenital anomalies.

## Discussion

Mankinen et al., [1] described 1q deletion syndrome and Surana et al., [26] described 4q duplication syndrome, and since then the advance in genomic research especially chromosome microarray (CMA) enabled many authors to discover the correlation between specific gene haploinsufficiency/duplication and certain phenotype in chromosome 1q deletion and 4q duplication. The use of CMA in detecting the copy number variances (CNVs) enables the discovery of new deletion/duplications syndromes. The American Academy of Pediatrics (AAP) recommended CMA to be the first tier in global developmental delay and intellectual disability (ID) [27].

The patient of the current study was presented to our department with developmental delay, dysmorphic features, microcephaly and hypogenesis of the corpus callosum.Thekaryotypes of the father and mother were normal. Consequently, the risk of recurrence is considered minimal if the chromosomal abnormality is de novo [28]. Chromosome 1q43q44 deletion syndrome and 4q32.1q35.2 duplication syndrome share many clinical features that have been reported in our patient. Both the syndromes are presented with MIC, hypertelorism, epicanthal fold, thick abnormal eyebrows, broad nasal bridge, low set malformed ears, long philtrum, small chin, short neck, single palmar crease, tapering fingers and clinodactyly [2–11, 17–21]. Chromosome 1 deletion is always with upward slanting of palpebral fissure which was found in the current patient. Chromosome 4q duplication differs as it is presented with downward slanting of palpebral fissure and sometimes prominent nasal bridge. The present patient has a normal heart and urogenital tract. The deleted 1q region in our previous patient reported with CHD and urogenital anomalies [7] was 10.4 Mb and the present patient had 6.5 Mb deletion, this indicates that CHD and urogenital tract abnormalities may be related to the proximal region on 1q43. Also, this is supported by the study of Nagmani et al., where the patients 3, 4 and 5 had CHD, those patients had microdeletion over proximal regions to the centromere, this indicate that the candidate gene for a heart defect and urogenital tract lies more proximal [14]. Also in partial 4q duplication some authors reported for congenital anomalies in the form of congenital heart defect, renal hypoplasia, neonatal cholestasia and chonal artesia [16, 18, 29–32], their duplicated 4q regions were more proximal than that reported in our patient. The 4q32.1q35.2 encompasses the HAND 2 gene which is essential for cardiac morphogenesis. Haploinsufficiency of HAND 2 may be the cause of the CHD and not the duplication. In the 4q duplication CHD is related to a more proximal region 4q22q23 [15–17].

The array CGH allowed defining the exactly involved cytobands and the encompassing genes, comparing the length of the deleted regions allowed to recognize the smallest region of overlap(SRO) and hence the correlation of the genotype/ phenotype.

The 1q43q44 region encompasses many important function genes (WWW.genecard.org): 1. CEP170 gene is expressed extensively in the brain. It encodes a functional protein which is a component of the centrosol complex. This complex is responsible for microtubules organization, neurogenesis and brain size. Mutations in the centrosol protein can cause MIC. 2. AKT3 (AKT Serine/Threonine Kinase 3) is a Protein-Coding gene. It plays a key role in regulating insulin signaling, cell survival, angiogenesis and tumor formation. 3. HNRNPU gene belongs to the subfamily of ubiquitously expressed heterogeneous nuclear ribonucleoproteins (hnRNPs). These are RNA binding proteins, and are associated with pre-mRNAs in the nucleus and control mRNA metabolism and transport. Depienne et al., confirmed that HNRNPU is the main gene involved in the seizures in patients with 1q43q44 deletion or gene mutation [11]. 4. ZBTB18 gene (previously ZNF238) encodes a C2H2-type zinc finger protein. It is involved in neuronal development. Authors correlated corpus callosum abnormalities (CCA) to the deletion of 1q43q44 encompassing ZBTB18 gene [12–14].

The 4q32.1q35.2 encompasses several genes important for brain and skull development (WWW.genecard.org): 1. HAND2 essential for cardiac morphogenesis and limb development. 2. GLRA3 plays an important role in the down-regulation of neuronal excitability. 3. GMP6A involved in the neuronal differentiation, including differentiation and migration of neuronal stem cells. The function of the genes involved in both 1q deletion and 4q duplication is very important for the brain and skull development and play similar or complementary functions. The similar phenotype of both 1q deletion and 4q duplication may reflect the function of the genes involved in brain and skull development.

MIC was reported in many patients with 1q43q44 deletion. Authors searched for the SRO that can cause MIC and several authors linked the haploinsufficiency of AKT3 gene as a cause for the MIC but not CCA [12–14]. Duplication involving AKT3 can cause macrocephaly [33]. A report on a boy who had a 4.1 Mb terminal deletion in 1q44 stated that the patient had MIC, the deletion encompasses the HNRNPU gene but not AKT3 [34]. A similar finding was reported on another patient who had a de novo 1.2 Mb interstitial deletion and had MIC, the deletion encompasses ZBTB18 and HNRNPU genes but not AKT3 [35]. The authors concluded that AKT3 causes a severe type of MIC. The MIC in their patients may be due to the effect of the deletion of HNTNPU or deletion of far regulatory elements that control the AKT3 gene [36–38]. Pure 4q32.14q35.2 is a rare syndrome and need more reporting to identify the SRO for MIC, some authors reported for MIC [15, 18], other for craniosynostosis and brachycephaly [20].

Depienne et al. [11] confirmed that the ZBTB18 is the main gene responsible for the CCA in patients with 1q terminal deletion. They also found the same abnormalities in three patients out of four who had ZBTB18 mutation [11]. This is not the case in all the patients with deletion or mutation in ZBTB18 gene as some had a normal corpus callosum, and the authors have related this to the incomplete penetrance of the gene [34].

van Bon et al. suggested the SRO for CCA to be 360 kb, and were the first to exclude AKT3 as a cause of CCA [6]. A report on 7 the patients with 1q deletion, which ranged from 0.08 to 4.35 Mb supported that CEP170 and ZNF238 (ZBTB18) were responsible for the CCA and excluded AKT3 as a cause of CCA and is responsible for MIC [14]. CCA was not found in association with the 4q duplication [39].

Terminal deletion 1q has been reported with moderate to severe ID, seizures, and nonspecific craniofacial anomalies. The sizes of the deletion ranging between 626Kb and 2.57 Mb and the size of SRO for ID and seizures as 188Kb encompass HNRNPU, FAM36A and NCRNA00201 genes which are the three candidate genes for nonsyndromic ID and seizures [13].

The 4 patients with 1q44 deletion, sharing 0.440 Mb which encompasses HNRPU, FAM36A and EFCAB2 all had delayed speech, seizures, and ID. The three patients out of 4 were reported to have CCA. The authors concluded that HNRPU affects the thickness of the corpus callosum and the other genes in 1q44 can affect CCA [36]. ID with variable degrees was reported in all the patients with a 1q43q44 deletion encompassing HNRNPU [6–11].

Hemming et al. summaries the finding in 159 patients with pure 1q43q44 deletion, they found 84% association between AKT3 and MIC, 83% association between CCA and deletion of the ZBTB18 gene, and 88.7% association between the HNRNPU gene deletion and the presence of seizure [40].

In a report on 17 patients with 1q43q44 microdeletions, in addition to the 37 patients who were reported before with 1q43q44 deletion, the authors focused on the three genes which are comprised of the most clinical picture of 1q43q44 deletion and are highly expressed in the brain: AKT3, HNRNPU and ZBTB18. They recognized an SRO of 1.36Mb that includes those selected genes [11]. They also reported the contribution of these three genes in neurodevelopmental delay [11]. These three candidate genes are responsible for neurodevelopmental disorders. They also compared the clinical data of the patients with microdeletion 1q43q44 to the patients with a point mutation in ZBTB18 and HNRNPU. They confirmed that AKT3 is responsible for MIC, ZBTB18 for CCA and HNRNPU for seizures [11, 41]. In the 4q duplication, the 4q33q34 is the critical region responsible for the development of the central nervous system and craniofacial structure. These critical regions involve the genes HAND2, GLR3 and GMB6A.

The prevalent phenotype observed in our patient was that of 1q43q44 deletion syndrome associated 4q duplication syndrome.

## Conclusion

We presented a patient with chromosome 1q43q44 deletion and 4q32.1q35.2 duplication. The aim was to correlate the genotype with the phenotype. We correlated the genotype/phenotype in our patient with pure 1q43q44 deletion and pure 4q32.1q35.2 duplication. We found that the involved genes on both the chromosomes have related functions in the brain and skull development, and this may be related to the network or common pathway of the functional genes and reflected in a similar phenotype. The deleted CEP170, AKT3, HNRNPU and duplicated HAND2, GLRA3, GMP6A are the candidate genes responsible for intellectual disability (ID), microcephaly (MIC), speech delay and dysmorphic facial features.. ZBTB18 was more involved in corpus callosum abnormalities (CCA). The urogenital abnormalities and CHD were suggested to be related to more proximal 1q43q44 deletion and 4q32.1q35.2 duplication. This is the first report on the combined effect of 1q43q44 deletion and 4q32.1q35.2 duplication and on the correlation of the genotype/phenotype of both the deletion and duplication.

The use of microarray delineated the presence of submicroscopic deletion or duplication, its size, genotype/phenotype correlation and identification of SRO of some clinical presentations like CCA, MIC and seizure. Patients represented with ID, seizures, MIC and CCA are candidates for the prediction of 1q43q44 microdeletion, and cytogenomic analysis is recommended for the micro-duplication of other chromosome. We recommended the use of HD array CGH in the patients presenting with CCA, MIC and ID.

## Abbreviations

CCA: Corpus callosum abnormalities
CGH: Comparative genomic hybridization
CHD: Congenital heart defect
CMA: Chromosome microarray
CNVs: Copy number variance
FISH: Fluorescence in situ hybridization
GTG: Gimsa trypsin G-banding
ID: Intellectual disability
ISCN: International system for Human Cytogenomic Nomenclature
MIC: Microcephaly
MLPA: Multiple ligation probe amplification
SD: Standard deviation
SRO: Smallest region of overlap
SZR: Seizure
WCP: Whole chromosome paint
